# Specializing Nurses as An Indirect Education Program for Stoma Patients

**DOI:** 10.3390/ijerph16132272

**Published:** 2019-06-27

**Authors:** Manuel García-Goñi

**Affiliations:** Department of Applied & Structural Economics and History, Universidad Complutense de Madrid, Campus de Somosaguas, 28223 Pozuelo de Alarcón, Madrid, Spain; mggoni@ucm.es

**Keywords:** patient education, program evaluation, specialist nurse, stoma care, cost-effectiveness

## Abstract

Education programs are beneficial for patients with different chronic conditions. Prior studies have examined direct education, where information is transferred directly to patients. In contrast, in this program, information is transferred directly to nurses who become specialists and transfer education individually to patients. Hence, this paper evaluates the impact of having specialist nurses for stoma patients at hospitals, as those nurses provide healthcare to patients but also inform and educate patients about their condition and needs. The analysis uses an observational study with ostomized patients in Spain at hospitals with and without specialist nurses, and measures health service utilization and health-related quality of life (HRQL), besides performing a cost analysis and a cost-effectiveness analysis at both types of hospitals. The results show that patients with access to specialist nurses self-manage better, present lower adverse events and a better evolution of HRQL, and significantly demand more consultations with specialist nurses and less to A&E, primary care or specialists, resulting in important savings for the health system. Consequently, specializing or hiring nurses to provide indirect education to stoma patients is cost-effective and highly beneficial for patients. This type of indirect education strategy might be considered for specific conditions with low incidence or difficulties in identifying target patients or delivering information directly to them.

## 1. Introduction

One of the main challenges in health management is the efficient allocation of resources aimed at obtaining improved health, or a better improvement of the health status of the population, for the money invested [1]. Education is one of the inputs in the health production function [2]. It leads to better health-related decisions and preventative behavior [3,4]. Education is particularly important for chronic patients. They need to learn how to live with their condition, which affects their quality of life, productivity and functional status [5]. Hence, it is recommended to engage with patients regarding the self-management of their disease to improve their quality of life and prevent or delay future health problems [6,7].

Patient education programs directly taught to patients have achieved positive results in terms of self-management for patients with, among others, asthma [8], cardiac disease [9,10], chronic obstructive pulmonary disease [11,12], type-2 diabetes [13] or melanoma [14]. Musekamp et al. [15] found that self-management programs have a positive effect not only on improving the quality of life of chronic patients but also in depressive symptoms. The direct patient education program has also been evaluated. Kristiansen and Antoft [16] showed the benefits of a group-based patient education program for patients with rheumatoid arthritis. For type-2 diabetes patients, Windrum et al. [17] found that a patient-centered model of education, promoting self-management and collaboration between patients and experts, results in better outcomes than a didactic program where patients passively receive standardized information. This type of patient-centered model of education, with direct interaction between patients and experts, has been explored and reviewed previously, as in Towle et al. [18], where the role of the active patient is considered in the education of health professionals.

Patient education is an integral part of the nursing process [19], with clinical nurses playing a key role in its delivery [20]. Friberg et al. [21] identifies the conditional factors for nurses’ patient education: beliefs and knowledge, educational environment, characteristics of healthcare organization, and interdisciplinary cooperation and collegial teamwork. They conclude that an improvement of the conditions under which nurses provide patient education is needed. Also, Bergh et al. [22] find that managerial barriers to the professional development of nurses’ patient education should be removed. Aiken et al. [23] evaluate the efficacy of nurses’ education. Using a sample of general hospitals in four states of the US, they estimate the relationship between nurse-to-patient staffing, organizational aspects, nurse education, and inpatient mortality in hospitals. They find that higher patient-to-nurse ratios increase the odds of patient deaths and failure-to-rescue, while better work environments and education in nurses decrease those odds.

The American Nurses Credentialing Center’s Commission on Accreditation [24] expresses the need to evaluate specific educational programs for nurses. This corresponds to the evaluation of specific indirect education programs to patients throughout the nursing profession, considering the individual characteristics of patients suffering from different chronic conditions.

This paper fills this gap by looking at the impact of education programs on chronic patients from a different angle: the effectiveness of education for specializing nurses in transferring information to patients. The case study is based on the implementation in hospitals of education programs for nurses, specializing them in the provision of care to recently ostomized patients; i.e., specialist in ostomy care (SOC) nurses. Coca et al. [25] followed an observational study approach in general hospitals in Spain to show how stoma patients with access to SOC nurses experienced significant improvements compared to patients without access to specialist nurses. Here, the same approach evaluates whether this program is cost-effective and results in economic savings for the health system, comparing the evolution of recently ostomized patients at hospitals with and without SOC nurses, taking into account the adverse events patients had to face and their health expenditures. This is relevant because nurses are playing an increasingly important role in most health systems, as part of the strategy to improve healthcare provision for chronic care [6,26]. Although there is a lack of information about the health expenditure devoted specifically to nursing care, the number of active professional nurses per 100,000 inhabitants has increased over recent years in many countries including Spain, from 51,044 in 2008 to 57,367 in 2016 [27]. Such evolution, given the regulation in wages in the public health sector in Spain, supposes an increase of health expenditure. Hence, this paper addresses the question of whether it is cost-effective and hospitals should, to some extent, program specific education to nurses to make them specialists in dealing with specific patients so as to provide more tailored healthcare and, at the same time, obtain savings for the healthcare system.

## 2. Materials and Methods

### 2.1. The Case Study: Stoma Patients

An ostomy is a surgical procedure in which an opening (stoma) is created for the discharge of body wastes towards an especially designed dispositive. Although there is no official registry of stoma patients in Spain, the prevalence is estimated to be 1.5 per 1000 inhabitants [28]; i.e., about 70,000 individuals and about 12,000 to 15,000 new cases annually [29].

Once a patient is ostomized, digestion works as for any other person, although the stoma becomes the way to eliminate wastes. Most body functions are unaffected and once adapted, patients live normal life with a few limitations, increasing their longevity. However, quality of life does not always improve. This surgery leaves a stoma instead of a scar, and the rupture of the body scheme produces a huge psychological impact [30,31,32].

Stoma patients need to maintain the hygiene of the stoma and correctly use the dispositive and drainable bag. To that effect, a learning process is recommended [33]. Although with time these tasks become easy, the first weeks after the ostomy are full of insecurities, adverse events, and health expenditures. The provision of education should help patients adapt quickly, accept their new condition, and prevent health complications. To that end, some hospitals provide education for nurses to become SOC nurses. The first international program of specific education for SOC nurses dates back to 1962 [34], discussing their role and utility [35,36,37].

The Spanish Society of SOC Nurses (*Sociedad Española de Enfermería Experta en Estomaterapia, SEDE*) was founded in 1988. Since then, the presence of SOC nurses has significantly increased. Currently, there are SOC nurses in all Spanish regions, although not in all hospitals. When this observational study took place, in 2013, 105 public hospitals (out of 220) had SOC nurses registered in Spain [29].

Hospitals with SOC nurses provide attention to stoma patients (or those scheduled to be ostomized) with the aim of easing their learning process; when there are no such patients, SOC nurses act as general nurses. Their health attention is individual, communicating on a one-to-one basis and considering the patient’s individual characteristics. At hospitals without SOC nurses, general nurses play this role. The implementation of this educational program to nurses aims to improve communication between nurses and patients, as well as their learning process and self-management.

### 2.2. The Design of the Sample

This paper uses an observational study containing registries of stoma patients gathered from 160 Spanish hospitals between March 2012 and May 2013. The sample is divided into two subsamples: Group I (treatment), with patients ostomized at hospitals with SOC nurses; Group II (control), with patients ostomized at hospitals without SOC nurses. Before the research was launched, a qualification of observational study not post-authorization (NO-EPA) was obtained from the Spanish Medicines and Health Products Agency (*Agencia Española del Medicamento y Productos Sanitarios*, AEMPS) and was approved by all the Research Ethics Committees of the corresponding health centers [38]. Two hundred and ninety-seven researchers participated in the collection of the information, and all Committees of Research Ethics approved the design. All patients with scheduled ostomies were included. They were all adults, could read and write, and provided written or oral consent to participate, with assured confidentiality and anonymity.

Three sets of information were collected. The first included socio-demographic, health status, and quality of life measures from both the time of the ostomy and three months afterwards. The second contained clinical information. The third was a follow-up questionnaire including information on adverse clinical events, demands of health provision, and use of dispositive and drainable bags at three different moments: first fortnight, second fortnight, and three months after the ostomy (for the second and third months separately). Clinical and life quality information was collected at the doctor consultation for patients in both groups. SOC nurses, working at the different hospitals in the sample, collected information regarding adverse events only for patients at their hospitals, which were enrolled in Group I. In contrast, information regarding adverse events for patients in Group II was gathered through telephone interviews, as there were no SOC nurses involved in those hospitals. The difference in the process of collecting information on adverse effects is assumed to be a limitation for the analysis, which might explain the lower response rate in Group II. Also, taken directly by SOC nurses, data on adverse events for patients in Group I might be more precise. However, in order to avoid bias, data gathered for patients in Group II took place via phone interviews at the same three moments in time so that patients would not forget their events or the way they solved them.

A total of 2200 questionnaires were sent and 908 were received (638—group I; 270—group II). However, 506 were rejected because either no ostomy was delivered or the quality of life information was incomplete. Complete information on quality of life was collected from 402 questionnaires (313—group I; 89—group II).

Table 1 shows the socio-demographic characteristics of those patients: 71.9% were males, and the average age was 61.8 years old (62.7—Group I; 59.3—Group II). Most patients were married (77.9%) and lived with their partner (80.9%). More than half had earned elementary education and were retired. An ostomy was programmed for 81.1% and an emergency for 18.9%. Programmed procedures were more common in Group I, whilst emergencies were more common in Group II [25]. Regarding the type of ostomy, 51.6% was colostomy, 28.7% ileostomy, and 16.5% urostomy. Only 3.2% of patients needed more than one ostomy. Colostomy was more common within group II, urostomy was equally frequent in both groups, and ileostomy was more frequent within group I [35]. Additionally, 31 patients from Group II were discarded because information on their adverse events or health utilization was incomplete. Hence, the final sample for the cost-effectiveness analysis consisted of 313 and 58 patients belonging to Groups I and II, respectively.

### 2.3. Health-Related Quality of life

HRQL is first measured through the Visual Analogue Scale (VAS) and the EuroQol-5D (EQ5D) index. VAS is a measure of perceived quality of life through the answer, in the range (0–100), to: “What is your health status today?”. Because VAS encompasses only one dimension, it might be biased. Thus, the EQ-5D index is commonly used [39,40]. Being complex and complete, it provides information on the self-perception of health status through five dimensions: mobility, self-care, usual activities, pain/discomfort, and anxiety/depression.

We measured the HRQL twice: right before the ostomy, and three months afterwards. Before the procedure, there should be no difference between patients at hospitals with or without SOC nurses. After the third month, the presence of a SOC nurse does not make any clinical difference, and all patients should have appropriately learned how to change the drainable bag and manage the dispositive [35]. Therefore, if there is any significant impact of stoma patients’ education through SOC nurses, it should be measured in the first three months after the ostomy.

The Mann–Whitney and Kolmogorov–Smirnov (*K-S*) tests (α = 0.05) were calculated using the software SPSS (IBM Corp., Armonk, NY, USA) at the time of the ostomy for VAS, indicating that both samples stem from different populations (*K-S Z* test = 2.1054) [38], which complicates any comparative analysis. However, results for the EQ5D index indicate that, before the ostomy, both samples stemmed from the same population (*K-S Z* test = 0.9324). Given those contradictory results, the analysis is validated through a third indicator of HRQL which is internationally accepted and specific for stoma patients, the Montreaux index [41]. The result indicates that, before the ostomy, both groups stemmed from the same population (*K-S Z* test = 1.0978). Therefore, the comparative analysis of the evolution in terms of HRQL for both samples is validated, and the instrument used for this exercise is the generally accepted EQ5D instrument.

### 2.4. Cost

The cost analysis differentiates between direct and indirect costs at three different moments: first fortnight, second fortnight (days 16 to 30), and second and third months (days 31 to 90) after the ostomy.

#### 2.4.1. Direct Costs

Direct costs consist of the purchase of ostomy supplies and the use of formal health provision. The purchase of supplies is registered in the follow-up questionnaire, including ostomy bags (one or two pieces), ostomy pastes and rings, adhesive or adhesive removers, skin protective barriers―such as powder, gels, wipes or cream—ostomy belts, and appliance cleansers. Their unit cost is obtained at the national reimbursement price list in 2013, the year of the observational study, from the Ministry of Health.

The follow-up questionnaire also registered clinical information on adverse events, including dermatitis, granulomas, oedema, haemorrhage, ischemia, necrosis, diarrhoea, tear, obstruction, prolapse, hernia, retraction, ulcer, dehiscence, stenosis, renal failure, and fistula, among others. The questionnaire keeps track of the health services demanded to solve adverse events: self-management, Accidents and Emergencies (A&E) departments, primary care, specialist care, SOC nurse care, general nurse care, pharmaceutical treatments, and inpatient stays. The cost of formal healthcare provision stems from utilization, multiplying the number of consultations of each type by its unit cost [42].

The calculations for direct costs have some limitations. First, some patients needed another surgical procedure in the period of analysis. However, information on the specific surgery is not available. All surgeries are assigned the same cost, which is the average cost of the most common surgeries within recently ostomized patients from the Catalogue by the Ministry of Health as a function of the Diagnosis-Related Groups. Similarly, the number of consultations with specialists is included in the dataset, without indicating which specific specialist patients visited. After corroborating with experts, this exercise assumed that all visits to specialists refer to surgeons, which was the most common visit.

The cost of an SOC nurse is assumed to be equal to that of a normal nurse, because the cost of their labor contract is identical. Furthermore, there is a cost in the provision of the educational program for nurses to become SOC nurses, which is key to this analysis. In Spain, those courses are mostly organized and financed by private companies, and the real cost for hospitals or nurses is zero. However, it is possible to find such programs on the Internet with costs from less than €100 to more than €200. Also, the cost of an official expert course of 20ECTS at a public university in Spain in the year of the analysis was €839.4 (€41.97 per ECTS). After the program, the SOC nurse can provide multiple consultations to many patients. In order to avoid undervaluing the cost of the educational program, a cost of €20 per visit to the SOC nurse was assigned in addition to the cost of a general nurse. Thus, if there is a bias in the analysis, it would be against SOC nurses, as it is expected that nurses will provide more than 42 consultations to stoma patients in their career (a greater number of consultations compensates the cost of the program of about €840). Finally, information on individual cost derived from pharmaceutical treatments is not available. After consultation with experts, those treatments are mostly anti-inflammatory and analgesic drugs. Given the high presence of generic products in those treatments, an average cost of €5 is assigned to pharmaceutical prescriptions.

#### 2.4.2. Indirect Costs

Indirect costs refer to the cost of other health-related needs by patients, not directly linked to the ostomy, as the assistance for usual activities or for anxiety. The analysis of indirect costs accounts for the productivity cost in the labor market by informal caregivers using three scenarios, in which the cost of one hour of informal or family care is €0, €6, or €10. In this way, the health system perspective in the cost-effectiveness analysis (only direct costs matter) is represented by the first scenario with no indirect costs. Two other scenarios are shown with plausible values for the cost of one hour of work for an unemployed person in Spain (€6 per hour) and for a domestic worker (€10 per hour).

The analysis does not account for productivity costs by stoma patients. Although in principle this could be considered as a limitation, this bias should be non-significant because of the patient’s average age (61.3 years), closeness to retirement, and their level of education.

At the same time, it is assumed that there is no differential cost before the ostomy. If there were, this should be related to the initial HRQL level. Finally, it is assumed that there is no selection bias because patients do not choose the hospital where they are ostomized.

### 2.5. Incremental Cost Effectiveness Analysis

The analysis shows the evolution of HRQL and incremental costs of recently ostomized patients at hospitals with and without SOC nurses. Without initial costs (before the ostomy), all registered costs are incremental. The incremental cost effectiveness ratio (ICER) is computed as the ratio of incremental cost to incremental HRQL, and the preference value for each health state is calculated following the time trade-off technique [43]. Hence, the analysis develops the tariff index for the HRQL indicator between 0 (death) and 1 (healthiest), allowing for the existence of negative values for worse-than-death evaluated health status. Then, acceptability curves are calculated for both groups [44].

## 3. Results

### 3.1. Clinical Adverse Events and Health Service Utilisation

Stata 12 (StataCorp LP, College Station, Texas, USA) was used for the descriptive analysis. Table 2 presents information on adverse events for stoma patients. Patients in Group I (with SOC nurses) present more adverse events in the first two weeks than patients in Group II (119 of 313―38.02%; 15 of 58―25.86%, respectively). Nevertheless, the occurrence of adverse events decreases in time much more significantly for patients in Group I: in the last period (months 2 and 3), only 15.34% of patients suffered adverse events in Group I, with this value being 24.14% in Group II. This result is aligned with the hypothesis of a better adaptation in patients with access to SOC nurses. Because there are patients with more than one adverse event, the total number of visits to health institutions is greater than the number of patients with adverse events.

Table 2 also shows how patients demand assistance to solve their adverse events. Group I patients show a reduced demand with time (292, 204, and 101 in the first, second and third period). In contrast, patients in Group II significantly increase their demand for health assistance in the last period (62, 32 and 94). Also, Group I patients mostly, and increasingly with time, demand health assistance from SOC nurses (66.10%, 74.51% and 90.10%). Taking into account the entire period of three months, consultations with the SOC nurse accounted for 73% of all consultations for Group I patients. In contrast, Group II patients trust self-management more in the first period (3% and 27% in Groups I and II, respectively) and need more and longer inpatient stays (10% and 22% in Groups I and II, respectively). In the second fortnight, patients in Group II use A&E more (28%) and have more frequent inpatient stays (34%). During the last period, Group II patients mostly demand consultations with specialists (63.83%).

### 3.2. Cost

The base scenario is that in which the cost of informal care hour is €6, although the tables also present the other two scenarios. Group I patients present a significantly lower average cost (Table 3), with total average costs for the three months of €642 (Group I) versus €1826 (Group II). Hence, this observational study estimates economic savings of 65% in healthcare provision to patients ostomized at hospitals with SOC nurses. The key to understanding the huge savings is in the first fortnight, when the average direct costs for patients with adverse events are €225 (Group I) and €1579 (Group II). Also, remarkably, the daily average costs in the three months are €7.14 (Group I) and €20.29 (Group II).

Most of the difference in cost is related to formal care, because the cost of ostomy supplies is not very different (€307 and €341, respectively) between groups. Within formal care, the main difference is a greater demand of health consultations with SOC nurses (Group I) instead of other, more expensive health services, such as hospital inpatient stays or A&E (Group II).

### 3.3. Health-Related Quality of Life and Economic Evaluation

The software EQIS 2.0 (Universidad Pública de Navarra, Pamplona, Spain) was used to estimate the economic evaluation exercise [45]. HRQL improved for all patients (Table 4), but significantly more in Group I. The average VAS increased from 64 to 78 (Group I) and from 64 to 66 (Group II). Regarding the EQ5D dimensions, the data show a better evolution in Group I, especially in usual activities. Following the Dolan index [40], the tariff goes from 0.733 to 0.814 (an increase of 0.08) in Group I and from 0.697 to 0.704 (an increase of 0.007) in Group II. Given the evolution of incremental costs (€642 and €1826, respectively), the ICER values are €7933 and €145,333 for Groups I and II, respectively. Hence, not only is the cost of healthcare provision much lower for patients at hospitals with SOC nurses, but their evolution in terms of quality of life is also better.

**Figure 1:**
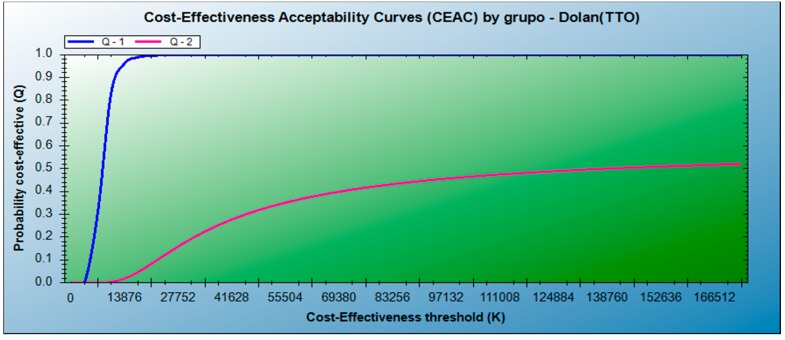
Cost-effectiveness acceptability curves for Groups I and II following the Dolan (TTO) methodology. Q-1, in blue, for Group I; Q-2, in pink, for Group II.

Providing education for nurses to become SOC nurses implies a cost per QALY (Quality Adjusted Life Years) significantly below €30,000, the threshold normally considered in Spain [46]. In contrast, not having SOC nurses results in a much greater cost per QALY. Figure 1 provides the sensibility analysis with acceptability curves. The likelihood of not rejecting having a SOC nurse as cost-effective (Group I) at a cost per QALY of €10,000 is greater than 90% (100% at a cost of €20,000). However, not having SOC nurses (Group II) is unlikely to be cost-effective. The results for Group I strictly predominate over the results for Group II.

## 4. Discussion

This paper performs a cost analysis and a cost-effectiveness analysis of an indirect education program for stoma patients by specialized nurses. It uses an observational study to compare the evolution in the HRQL and healthcare cost of recently ostomized patients during the three months after the procedure in hospitals with SOC nurses (Group I) and without them (Group II). A better evolution of daily activities, lack of pain and lack of anxiety is found in patients with access to SOC nurses, supporting patients to develop better self-confidence and manage their condition right after the ostomy, when they need to adapt to their new condition.

Clinical health status follows the same evolution with fewer adverse events for patients in Group I, resulting in a lower demand of health services and thus a lower incremental cost. Hence, this analysis indicates that having SOC nurses at hospitals is cost-effective because they improve the transmission of information to stoma patients and their self-management. The cost of the education program is very low and only increases the average incremental cost of patients by €28, while its related increase in HRQL is significant. This result remains the same when departing from different assumptions on the cost of informal care.

There are some limitations of this analysis. It is an observational study which is not randomized, with different rate of responses in both groups and design of data collection. There might also be unobserved variables affecting the results. Consequently, any conclusions have to be considered with caution. However, the data stems from a wide number of hospitals within the Spanish NHS.

This work is novel in that it presents an economic evaluation of indirect patient education programs, measuring the differential value of specialized nurses. The results are robust and show economic savings derived from the cost-effective use of SOC nurses. Patients behave differently when SOC nurses are available, increasing their consultations with them for solving problems, feeling more confident, self-managing their condition better, and reducing the frequency of adverse events. Furthermore, patients with access to SOC nurses drastically reduce their demand for other (and more expensive) health services as consultations to specialists, or A&E, and as a result, there is a more efficient use of healthcare resources and a much lower level of health expenditure.

## 5. Conclusions

Chronic patients, as stoma patients, must be informed and learn how to self-manage their condition to avoid unnecessary adverse events and health expenditure. This paper shows that this information is satisfactorily transmitted by SOC nurses, and that this indirect education program is cost-effective. Furthermore, having SOC nurses at hospitals accelerates patients’ adaptation.

This work contributes to the extensive literature claiming a better transmission of information to chronic patients, permitting them to learn to self-manage their condition and become themselves the first step of healthcare provision, as well as to be able to prevent further future health problems, resulting in economic savings. Specialist nurses, at least in the case of SOC nurses for stoma patients, are cost-effective in obtaining this goal.

Patient education programs are effective for many chronic conditions. For those conditions, the direct education of patients is a good strategy to improve their knowledge about their condition, their lifestyles, and thus their quality of life. Still, there might be other chronic conditions with a lower expected number of patients, which are more difficult to identify, or which pose greater difficulties in delivering an education program, for which direct education of patients is difficult to organize or expensive. For patients suffering from those conditions, this paper points to indirect education programs performed by specialist nurses. They, at least in the case of stoma patients, provide more tailored care to patients, are successful in transmitting the information patients need to manage, and represent a cost-effective alternative for improving patients’ health status and producing savings for the health system. The research findings highlight the need for more research on this topic.

## Figures and Tables

**Table 1:** Socio-demographic characteristics in the sample. SOC: specialist in ostomy care.

Descriptive Variable	Group I Hospitals with SOC Nurses	Grupo II Hospitals without SOC Nurses	Total Sample
*N*	313	89	392
Average age in years (std. dev.)	62.7 (13.1)	59.3 (14.2)	61.8 (13.7)
Marital status			
Married	80.8%	74.5%	77.9%
Single	10.3%	13.8%	11.9%
Widow	6.1%	4.3%	5.2%
Other	2.8%	7.4%	5.0%
Living situation			
With partner and children	48.8%	48.4%	48.6%
With partner without children	33.0%	31.4%	32.3%
Alone	8.8%	7.4%	8.2%
With parents	6.0%	3.7%	5.0%
With friends	0.6%	2.7%	1.5%
Other	2.8%	6.4%	4.5%
Level of studies			
Elementary	48.6%	58.3%	52.8%
High school	29.0%	23.9%	26.8%
University level studies	12.9%	16.6%	14.5%
Other	9.5%	1.2%	5.9%
Employment situation			
Pensioner	54.5%	47.0%	50.8%
Employed	27.6%	38.7%	33.1%
Unemployed	6.5%	3.9%	5.3%
Retired (disabled)	3.3%	2.8%	3.0%
Student	0.9%	2.2%	1.5%
Other	7.0%	5.5%	6.3%

**Table 2:** Adverse events in stoma patients and demand of health services to solve them.

Descriptive Variable	First Fortnight				Second Fortnight				Second and Third Months				Entire Period: Three Months			
	Group I Patients at Hospitals with SOC (*N* and %)		Group II Patients at Hospitals without SOC (*N* and %)		Group I Patients at Hospitals with SOC (*N* and %)		Group II Patients at Hospitals without SOC (*N* and %)		Group I Patients at Hospitals with SOC (*N* and %)		Group II Patients at Hospitals without SOC (*N* and %)		Group I Patients at Hospitals with SOC (*N* and %)		Group II Patients at Hospitals without SOC (*N* and %)	
Patients with and without adverse events																
With adverse events	119	38.02%	15	25.86%	79	25.24%	10	17.24%	48	15.34%	14	24.14%				
Without adverse events	194	61.98%	43	74.14%	234	74.76%	48	82.76%	265	84.66%	44	75.86%				
*N*	313		58		313		58		313		58					
Visits to each type of formal health assistance for patients with adverse events																
Self-management	10	3.42%	17	27.42%	8	3.92%	7	21.88%	4	3.96%	1	1.06%	22	3.69%	25	13.30%
Accidents & Emergencies	5	1.71%	2	3.23%	6	2.94%	9	28.13%	0	0.00%	12	12.77%	11	1.84%	23	12.23%
Primary Care doctor	2	0.68%	2	3.23%	2	0.98%	3	9.38%	1	0.99%	2	2.13%	5	0.84%	7	3.72%
Specialist	12	4.11%	3	4.84%	10	4.90%	1	3.13%	4	3.96%	60	63.83%	26	4.36%	64	34.04%
SOC nurse	193	66.10%	3	4.84%	152	74.51%	0	0.00%	91	90.10%	8	8.51%	436	73.03%	11	5.85%
Nurse	38	13.01%	16	25.81%	12	5.88%	0	0.00%	1	0.99%	0	0.00%	51	8.54%	16	8.51%
Pharmaceutical prescription	0	0.00%	5	8.06%	0	0.00%	1	3.13%	0	0.00%	0	0.00%	0	0.00%	6	3.19%
Hospital (days of inpatient stay)	32	10.96%	14	22.58%	14	6.86%	11	34.38%	0	0.00%	11	11.70%	46	7.71%	36	19.15%
Total Consultations	292	100%	62	100%	204	100%	32	100%	101	100%	94	100%	597	100%	188	100%

**Table 3:** Costs of stoma patients in groups I and II.

Type of Cost	Costs in the First Fortnight					
	Group I			Group II		
	All Patients	Patients without Adverse Events in the First Fortnight	Patients with Adverse Events in the First Fortnight	All Patients	Patients without Adverse Events in the First Fortnight	Patients with Adverse Events in the First Fortnight
	*N* = 313	*N* = 194	*N* = 119	*N* = 58	*N* = 43	*N* = 15
Cost of ostomy supplies	54.90	49.63	63.49	50.98	52.18	47.52
Cost of formal health assistance	86.73	40.49	162.11	397.48	1.87	1531.57
Direct costs	141.63	90.12	225.61	448.46	54.05	1579.09
Indirect costs	67.34	57.52	83.34	222.06	181.02	339.71
Total costs (assuming cost of informal care hour of €6)	208.97	147.64	308.94	670.52	235.08	1918.80
Average daily cost	13.93	9.84	20.60	44.70	15.67	127.92
Educational program cost	12.33	0.00	32.44	0.00	0.00	0.00
	**Costs in the first and second fortnight (first month after ostomy)**					
	Group I			Group II		
	All patients	Patients without adverse events in the second fortnight	Patients with adverse events in the second fortnight	All patients	Patients without adverse events in the second fortnight	Patients with adverse events in the second fortnight
	*N* = 313	*N* = 234	*N* = 79	*N* = 58	*N* = 48	*N* = 10
Cost of ostomy supplies	107.41	101.40	125.21	104.71	103.12	112.33
Cost of formal health assistance	128.57	57.27	339.55	503.79	465.36	684.64
Direct costs	235.98	158.67	464.76	607.87	568.48	796.97
Indirect costs	102.40	86.78	148.65	354.85	346.43	395.29
Total costs (assuming cost of informal care hour of €6)	338.38	245.46	613.40	962.73	914.91	1192.26
Average daily cost	11.28	8.18	20.45	32.09	30.50	39.74
Educational program cost	22.04	7.35	65.57	0.00	0.00	0.00
	**Costs in the first three months after the ostomy**					
	Group I			Group II		
	All patients	Patients without adverse events in months 2 and 3	Patients with adverse events in months 2 and 3	All patients	Patients without adverse events in months 2 and 3	Patients with adverse events in months 2 and 3
	*N* = 313	*N* = 265	*N* = 48	*N* = 58	*N* = 44	*N* = 14
Cost of ostomy supplies	307.06	302.73	331.02	341.88	350.50	314.78
Cost of formal health assistance	160.00	112.24	422.93	673.30	425.46	1452.23
Direct costs	466.95	414.97	753.94	1015.18	775.96	1767.02
Indirect costs	175.69	132.92	411.80	811.27	534.24	1681.92
Total costs (assuming cost of informal care hour of 6€)	642.75	547.89	1165.74	1826.45	1310.20	3448.94
Average daily cost	7.14	6.09	12.95	20.29	14.56	38.32
Educational program cost	27.86	16.91	88.33	0.00	0.00	0.00
Total costs (assuming cost of informal care hour of 0€)	507.30			1374.17		
Total Costs (assuming cost of informal care hour of 10€)	732.86			2127.97		

**Table 4:** Evolution of the health-related quality of life (HRQL) and incremental cost effectiveness ratio (ICER) in stoma patients. VAS: Visual Analogue Scale.

	Group I (*N* = 313)						Group II (*N* = 58)					
	Before Ostomy		Three Months after the Ostomy		Variation		Before Ostomy		Three Months after the Ostomy		Variation	
	*N*	%	*N*	%	*N*	%	*N*	%	*N*	%	*N*	%
Dimensions in the HRQL												
Mobility												
I have no problems in walking about	273	87.2%	273	87.2%	0	0.0%	48	82.8%	44	75.9%	−4	−6.9%
I have some problems in walking about	36	11.5%	37	11.8%	1	0.3%	8	13.8%	13	22.4%	5	8.6%
I am confined to bed	4	1.3%	3	1.0%	−1	−0.3%	2	3.4%	1	1.7%	−1	−1.7%
Self-Care												
I have no problems with self-care	289	92.3%	279	89.1%	−10	−3.2%	48	82.8%	46	79.3%	−2	−3.4%
I have some problems washing or dressing myself	18	5.8%	29	9.3%	11	3.5%	7	12.1%	11	19.0%	4	6.9%
I am unable to wash or dress myself	6	1.9%	5	1.6%	−1	−0.3%	3	5.2%	1	1.7%	−2	−3.4%
Usual activities												
I have no problems with performing my usual activities	262	83.7%	248	79.2%	−14	−4.5%	43	74.1%	31	53.4%	−12	−20.7%
I have some problems with performing my usual activities	40	12.8%	57	18.2%	17	5.4%	11	19.0%	22	37.9%	11	19.0%
I am unable to perform my usual activities	11	3.5%	8	2.6%	−3	−1.0%	4	6.9%	5	8.6%	1	1.7%
Pain/Discomfort												
I have no pain or discomfort	179	57.2%	234	74.8%	55	17.6%	31	53.4%	27	46.6%	−4	−6.9%
I have moderate pain or discomfort	115	36.7%	75	24.0%	−40	−12.8%	20	34.5%	29	50.0%	9	15.5%
I have extreme pain or discomfort	19	6.1%	4	1.3%	−15	−4.8%	7	12.1%	2	3.4%	−5	−8.6%
Anxiety/Depression												
I am not anxious or depressed	168	53.7%	227	72.5%	59	18.8%	37	63.8%	33	56.9%	−4	−6.9%
I am moderately anxious or depressed	117	37.4%	80	25.6%	−37	−11.8%	21	36.2%	24	41.4%	3	5.2%
I am extremely anxious or depressed	28	8.9%	6	1.9%	−22	−7.0%	0	0.0%	1	1.7%	1	1.7%
HRQL through VAS												
Mean	64.278		78.099		13.821		64.310		66.719		1895	
Standard deviation	23.230		14.887		25.734		23.235		18.420		22.045	
Estimated HRQL tariff												
Mean	0.733		0.814		0.081		0.697		0.704		0.007	
Standard deviation	0.015		0.011				0.039		0.030			
Incremental Cost (cost of informal care hour 0€)												
Mean (standard deviation)	597.30 (740.49)						1374.17 (2224.06)					
Incremental Cost (cost of informal care hour 6€)												
Mean (standard deviation)	642.75 (1197.56)						1826.45 (2311.52)					
Incremental Cost (cost of informal care hour 10€)												
Mean (standard deviation)	732.86 (1614.92)						2127.97 (2484.96)					
ICER (€/QALY)												
Total cost (cost of informal care hour 0€)	6262.96						106,916.67					
Total cost (cost of informal care hour 6€)	7933.70						145,333.33					
Total cost (cost of informal care hour 10€)	9047.65						170,833.33					
